# NDRG2 Expression Correlates with Neurofibrillary Tangles and Microglial Pathology in the Ageing Brain

**DOI:** 10.3390/ijms21010340

**Published:** 2020-01-04

**Authors:** Motaz M. Fadul, Claire J. Garwood, Rachel Waller, Navonna Garrett, Paul R. Heath, Fiona E Matthews, Carol Brayne, Stephen B. Wharton, Julie E. Simpson

**Affiliations:** 1 Department of Neuroscience, Sheffield Institute for Translational Neuroscience, University of Sheffield, Sheffield S10 2HQ, UK; mmfadul1@sheffield.ac.uk (M.M.F.); C.Garwood@sheffield.ac.uk (C.J.G.); r.waller@sheffield.ac.uk (R.W.); ngarrett1@sheffield.ac.uk (N.G.); p.heath@sheffield.ac.uk (P.R.H.); s.wharton@sheffield.ac.uk (S.B.W.); 2 Population Health Sciences Institute, University of Newcastle, Newcastle NE4 5PL, UK; Fiona.Matthews@newcastle.ac.uk; 3 Department of Public Health and Primary Care, Institute of Public Health, University of Cambridge, Cambridge CB2 0SR, UK; cb105@medschl.cam.ac.uk

**Keywords:** N-myc downstream regulated gene 2 (NDRG2), astrocyte, ageing brain, neurofibrillary tangles

## Abstract

Astrocytes play a major role in the pathogenesis of a range of neurodegenerative diseases, including Alzheimer’s disease (AD), undergoing dramatic morphological and molecular changes that can cause potentially both beneficial and detrimental effects. They comprise a heterogeneous population, requiring a panel of specific phenotype markers to identify astrocyte subtypes, changes in function and their relation to pathology. This study aimed to characterise expression of the astrocyte marker N-myc downstream regulated gene 2 (NDRG2) in the ageing brain, investigate the relationship between NDRG2 and a panel of astrocyte markers, and relate NDRG2 expression to pathology. NDRG2 specifically immunolabelled the cell body and radiating processes of astrocytes in the temporal cortex of the Cognitive Function and Ageing Study (CFAS) neuropathology cohort. Expression of NDRG2 did not correlate with other astrocyte markers, including glial fibrillary acidic protein (GFAP), excitatory amino acid transporter 2 (EAAT2) and glutamine synthetase (GS). NDRG2 showed a relationship to AT8^+^ neurofibrillary tangles (*p* = 0.001) and CD68^+^ microglia (*p* = 0.047), but not β-amyloid plaques or astrocyte nuclear γH2AX immunoreactivity, a marker of DNA damage response. These findings provide new insight into the astrocyte response to pathology in the ageing brain, and suggest NDRG2 may be a potential target to modulate this response.

## 1. Introduction

While much of the focus in dementia research has been on the neuron, other cell types are also important. Astrocytes, the most abundant cell type in the central nervous system (CNS), play an essential role in homeostasis, including neuronal support, synapse formation and function, maintenance and metabolism [1,2]. They are classified into two major groups on the basis of their morphology and anatomical location; the fibrous astrocytes of the white matter and the protoplasmic astrocytes of the grey matter [2]. Astrocytes comprise a heterogeneous population [3,4], and different markers may reflect different functional and structural aspects of the astrocyte, and their relationship to pathology.

Disruption of the normal astrocyte–neuronal interaction can lead to synaptic dysfunction and contribute to cognitive impairment, as recently reviewed [5]. Production of pro-inflammatory cytokines and loss of neuronal support, such as neurotransmitter recycling, may contribute to neurodegeneration. As part of the tripartite synapse, astrocytes regulate levels of neurotransmitters, including the excitatory transmitter glutamate, and play a major role in preventing excitotoxicity caused by the accumulation of excess glutamate [6,7]. Astrocytes remove excess glutamate from the extracellular space via excitatory amino acid transporter 2 (EAAT2), and contain the enzyme glutamine synthetase (GS) which converts glutamate to glutamine [8]. In addition to regulating neurotransmission, astrocytes are responsible for providing metabolic support to neurons via the lactate shuttle [9].

Astrocytes react to a variety of CNS pathologies, including age-associated neurodegenerative diseases such as Alzheimer’s disease (AD), by upregulating expression of the intermediate filament glial fibrillary acidic protein (GFAP). Astrogliosis is one of the earliest changes in response to accumulating AD pathology, β-amyloid (Aβ) plaques and neurofibrillary tangles (NFT) of hyperphosphorylated tau [10]. This reactive astrogliosis is considered to play a neuroprotective role, protecting against oxidative stress and promoting the clearance of Aβ [11].

N-myc downstream regulated gene 2 (NDRG2) mRNA and its corresponding protein are principally expressed by protoplasmic and fibrous astrocytes throughout the brain [12,13,14]. Increased expression of NDRG2 has been reported in neurodegenerative diseases, including AD [15,16], frontotemporal lobar degeneration [17] and stroke [18]. The exact functions of NDRG2 are unknown but it has been shown to have an inhibitory effect on tumour proliferation [19,20], reduce production of reactive oxygen species and apoptosis [18], facilitate the hyperphosphorylation of tau [16], protect blood–brain barrier integrity [21], and facilitate the uptake of glutamate by astrocytes [22].

The Cognitive Function and Ageing Neuropathology Study (CFANS) is a large scale, population representative neuropathology cohort (>65 y) which contains the spectrum of age-associated neurodegenerative pathologies. The study combined longitudinal assessment of cognitive function, including Mini-Mental State Examination (MMSE), with a brain collection program to enable neuropathological associations with cognitive impairment to be determined without preselection into clinical groups [23]. The current study aimed to characterise the expression of NDRG2, investigate the relationship between NDRG2 and a panel of astrocyte markers (GFAP, EAAT2, GS), and relate the expression of NDRG2 to age-associated neurodegenerative pathology in the CFAS cohort.

## 2. Results

### 2.1. Comparison of the Immunoreactive Profile of NDRG2 with a Panel of Astrocyte Markers

**Figure 1:**
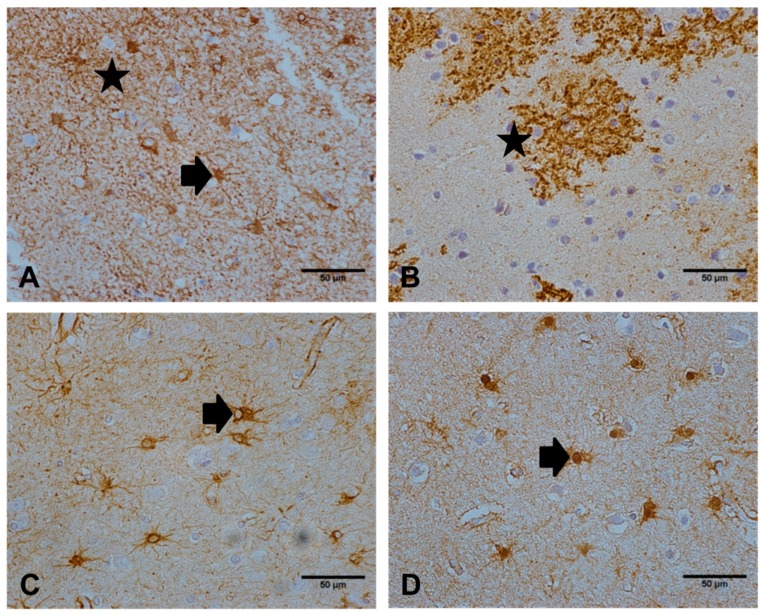
Immunoreactive profiles observed with a panel of astrocyte markers. (**A**) N-myc downstream regulated gene 2 (NDRG2 immunolabelled the astrocyte cell body (as indicated by the arrow) and radiating processes (star). (**B**) excitatory amino acid transporter 2 (EAAT2) extensively immunolabelled the extending processes (star) of astrocytes. Both (**C**) glial fibrillary acidic protein (GFAP) and (**D**) glutamine synthetase (GS) predominantly stained astrocyte cell bodies and immediate cell processes (arrow). Images are representative of the astrocyte immunolabelling profile observed in the temporal cortex of 97 cases. Scale bar represents 50 μm.

NDRG2 exclusively immunolabelled the astrocyte cell body and radiating processes throughout the cortex (Figure 1A). Both NDRG2 and EAAT2 (Figure 1B) extensively immunolabelled the distal delicate processes of astrocytes, in comparison to antibodies against GFAP (Figure 1C) and GS (Figure 1D), which predominantly stained astrocyte cell bodies and proximal cell processes. Quantitative assessment of the immunoreactive profile of NDRG2, GFAP, GS, and EAAT2 across the cohort is shown in Table 1 and Figure 3A. Significant differences between these measures of astrocytes were detected (Friedman’s *p* < 0.001). NDRG2 immunoreactivity did not correlate with either brain pH (r_s_ = −0.067, *p* = 0.562) or PMD (r_s_ = 0.03, *p* = 0.79).

### 2.2. NDRG2 Does Not Correlate with the Expression of Other Astrocyte Markers

Expression of NDRG2 did not correlate with levels of GFAP (r_s_ = −0.155, *p* = 0.134), EAAT2 (r_s_ = −0.178, *p* = 0.091) or GS (r_s_ = 0.085, *p* = 0.429). In contrast, GFAP negatively correlated with EAAT2 expression (r_s_ = −0.249, *p* = 0.015) and EAAT2 expression correlated with GS expression (r_s_ = 0.209, *p* = 0.046) in the ageing brain. GFAP expression did not correlate with GS (r_s_ = 0.147, *p* = 0.163).

### 2.3. Not All Astrocytes are NDRG2^+^

**Figure 2:**
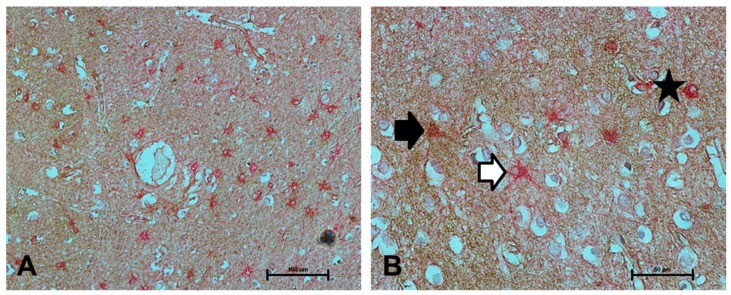
Colocalisation of NDRG2 with GFAP. (**A**–**B**) Colocalisation of NDRG2 (brown) with GFAP (red) was frequently observed (as indicated by the star), however both NDRG2+/GFAP- (black arrow) and NDRG2-/GFAP+ astrocytes (white arrow) were detected in the ageing temporal cortex. Scale bar represents 100 μm in A and 50 μm in B.

To assess if NDRG2 immunolabelled a greater proportion of astrocytes than the current standard astrocyte marker GFAP, dual labelling was performed. While co-localisation of NDRG2 with GFAP was frequently observed, both NDRG2^+^/GFAP^−^ and NDRG2^−^/GFAP^+^ astrocytes were detected (Figure 2).

### 2.4. NDRG2 Expression Correlates with Local Tau Pathology

**Figure 3:**
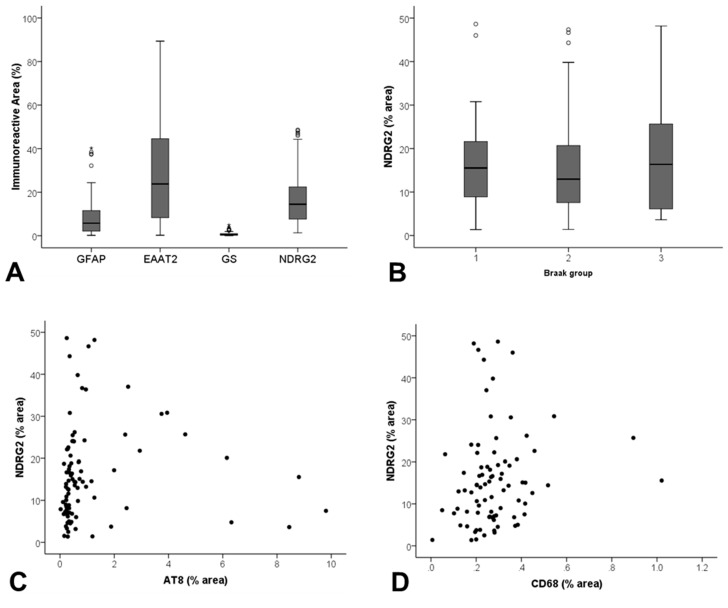
Assessment of NDRG2 expression in the ageing temporal cortex. (**A**) Significant differences in the immunoreactive profile of a panel of astrocyte markers were detected (*p* < 0.001). (**B**) NDRG2 expression did not correlate with Braak stage, but did show a relationship to (**C**) local levels of tau pathology (AT8) (r_s_ = 0.335, *p* = 0.001), and (**D**) CD68^+^ microglia (r_s_ = 0.219, *p* = 0.047).

NDRG2 expression did not correlate with Braak NFT stage, a measure of global AD pathology in the ageing brain (JT *p* = 0.786) (Figure 3B), in contrast to both GFAP (JT *p* = 0.032) and GS (JT *p* = 0.017) which showed a positive correlation.

**Figure 4:**
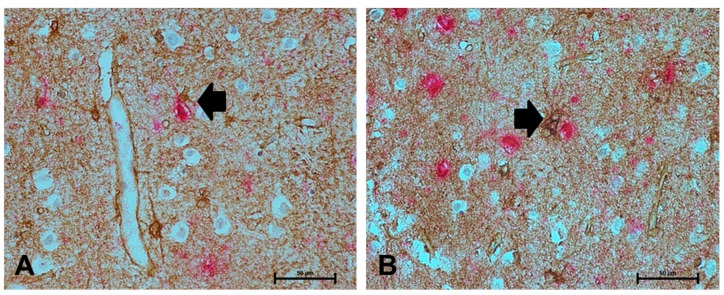
NDRG2^+^ astrocytes associate with neurofibrillary tangles (NFT) in the ageing brain. (**A**–**B**) NDRG2^+^ astrocytes (brown) associated with AT8 immunoreactivity (red), as indicated by the black arrow. Scale bar represents 50 μm.

As astrocytes respond to pathology in their immediate vicinity, we investigated the relationship of NDRG2 to local measures of AD pathology, assessed by Aβ and AT8^+^% area immunoreactivity. NDRG2 expression did not correlate with Aβ (r_s_ = 0.154, *p* = 0.146), but showed a relationship to AT8 (r_s_ = 0.335, *p* = 0.001) (Figure 3C). Dual immunolabelling confirmed increased expression of NDRG2 by astrocytes in the vicinity of AT8^+^ neurones (Figure 4).

GS expression correlated with both Aβ (r_s_ = 0.244, *p* = 0.02) and AT8 (r_s_ = 0.242, *p* = 0.023). Neither GFAP nor EAAT2 expression correlated with local levels of Aβ (GFAP r_s_ = 0.128, *p* = 0.221; EAAT2 r_s_0.071, *p* = 0.503) or AT8 (GFAP r_s_ = 0.163, *p* = 0.124; EAAT2 r_s_ = −0.18, *p* = 0.087).

### 2.5. NDRG2 Expression Correlates with CD68 but not with Oxidative DNA Damage

To determine if NDRG2 associated with microglial reactivity we investigated the relationship with CD68 and MHC II. NDRG2 correlated with CD68 (r_s_ = 0.219, *p* = 0.047) (Figure 3D), but not MHC II expression (r_s_ = 0.164, *p* = 0.146) in the ageing brain. No other astrocyte marker correlated with either CD68 (GFAP r_s_ = 0.09, *p* = 0.409; EAAT2 r_s_ = −0.051, *p* = 0.638; GS r_s_ = 0.193, *p* = 0.078) or MHC II (GFAP r_s_ = −0.177, *p* = 0.109; EAAT2 r_s_ = −0.013, *p* = 0.904; GS r_s_ = 0.109, *p* = 0.331).

To determine if NDRG2 associated with oxidative stress we investigated their relationship with γH2AX, a marker of oxidative DNA damage. NDRG2 did not correlate with astrocyte nuclear γH2AX immunoreactivity (r_s_ = 0.179, *p* = 0.089). Similarly, neither GFAP (r_s_ = 0.025, *p* = 0.813), nor GS (r_s_ = 0.14, *p* = 0.183) correlated with γH2AX, however EAAT2 correlated with increased levels of astrocyte nuclear γH2AX immunoreactivity (r_s_ = 0.222, *p* = 0.033).

## 3. Discussion

NDRG2 is expressed at high levels in the brain, and plays a role in growth, differentiation and development, and the stress response [24]. NDRG2 is upregulated in the hippocampus in AD in post-mortem studies [15] and animal models [16]. However, in contrast to these studies which report that NDRG2 expression is associated with neurons, the current study demonstrates NDRG2 is exclusively associated with astrocytes in the ageing brain and correlates with tau and microglial pathology.

NDRG2 was first identified in a normal human brain cDNA library in 2001 [25]. While some studies report NDRG2 expression in pyramidal neurons, senile plaques and dystrophic neurons in the hippocampus in AD [15], more recent studies investigating the expression of NDRG2 in a variety of mammalian brains, including human, demonstrate that NDRG2 is a specific marker for astrocytes and co-localises with the majority of known astrocyte markers [13,14]. In the current study, significant differences in the immunoreactive profiles of the panel of astrocyte markers were detected and the patterns of staining varied greatly across the cohort, supporting the findings of a recent study comparing NDRG2 expression with other astrocyte markers in different mouse cerebral regions [26]. Both GFAP and GS predominantly labelled astrocyte cell bodies and immediate cell processes. However, as only approximately 15% of the total astrocyte volume is GFAP^+^, the entire astrocyte domain is not fully visualised [27]. In contrast, antibodies to NDRG2 and EAAT2 immunolabel the extending radiating processes of astrocytes clearly demonstrating the extent of the astrocyte domain.

Levels of NDRG2 increase in AD transgenic mice (APP/PS1), and suppression of NDRG2 has been shown to improve cognitive function in this model [28]. As the NDRG2^+^ distal processes of astrocytes are in close proximity to synapses, this suggests a role in impairing synaptic transduction [13]. In contrast, NDRG2 deficiency has recently been shown to exacerbate long-term memory impairment in this model of AD, suggesting a neuroprotective role for NDRG2 [29]. Recent studies have shown that knockdown of NDRG2 significantly reduces tau phosphorylation in a human neuroblastoma cell line overexpressing wild type APP695 [16]. Our data demonstrate that expression of NDRG2 positively correlates with AT8^+^ tau pathology in the ageing brain; however, whether this is a mechanism actively contributing to neuronal pathology or a response to neurodegeneration is currently unknown.

*NDRG2* is an injury response gene that positively regulates early astrocyte activation and the inflammatory response in a mouse model of cortical stab injury [30]. The reciprocal interactions between microglia and astrocytes play a key role in the progression of AD [31]. In the current study NDRG2 did not correlate with expression levels of the other astrocyte markers but did correlate with CD68, a lysosomal protein present in phagocytic microglia in the ageing brain. CD68^+^ microglia positively associate with dementia, neuritic plaques and tangles in the ageing population [32]. Whether NDRG2^+^ astrocytes are linked to microglial pathology, or vice-versa, is unknown and whether these NDRG2^+^ glia are adopting a neuroprotective or a neurotoxic phenotype needs to be determined. Additional studies to interrogate the association with other microglial markers, including TREM−2, are also required.

NDRG2 is mainly expressed in the cytoplasm, however it has been shown to translocate to the nucleus under stress conditions [33,34,35], and control astrocyte morphology via Rho-GTPase signalling [36]. Oxidative stress and the associated DNA damage may directly impact astrocytes, consequently modifying their normal function [37]. We previously reported a reduction in expression of γH2AX by astrocytes associated with increasing levels of Alzheimer’s type pathology [38]. Here our results demonstrate that the astrocyte DNA damage response does not correlate with NDRG2 but does correlate with expression of EAAT2. Both γH2AX and EAAT2 were highest in cases with low levels of AD pathology, and which may constitute a stress-response aimed at increasing glutamate uptake and reducing neuronal excitotoxicity at the earliest stages of AD pathology.

Ageing population-representative neuropathology cohorts, such as CFAS, are a valuable resource for investigating correlates of cellular pathology. A comprehensive panel of astrocyte markers, including NDRG2, is required to fully elucidate the complexity of the astrocyte response, providing a snapshot of different aspects of astrocyte pathobiology and how they relate to age-related neurodegenerative pathology. In the current study we demonstrate that NDRG2 is exclusively expressed by astrocytes, and that levels of NDRG2 positively correlate with neurofibrillary tangles and CD68^+^ microglial pathology in the temporal cortex of the CFAS cohort. Future studies are required to characterise the interaction between NDRG2^+^ astrocytes, microglia and tangle pathobiology, and may identify potential therapeutic targets to modify the astrocyte phenotype.

## 4. Materials and Methods

### 4.1. Human CNS Cases

Human autopsy brain tissue was obtained from one centre (Cambridge, 97 cases) of the Cognitive Function and Ageing Study (CFAS) [23,39], following multi-centre research ethics committee (REC) approval (REC Reference number 15/SW/0246, 10 August 2015). Formalin-fixed lateral temporal cortex samples (superior/middle temporal gyrus, Brodmann areas 22/21) of all cases in the subcohort were used to maintain the population-representative basis of the study. Neuropathological lesions were previously assessed as part of the core CFAS neuropathology study using a modified protocol from the Consortium to Establish a Registry of Alzheimer’s Disease (CERAD) [40] (wwws.cfas.ac.uk) and Braak neurofibrillary tangle (NFT) staging [41]. The mean age of death was 85.6 years (SEM 7.4). The median post-mortem delay was 17 h (interquartile range [IQR] 10–32 h) and brain pH 6.49 (IQR 6.25–6.75).

### 4.2. Immunohistochemistry

Immunohistochemistry was performed using a standard avidin-biotin complex (ABC) method. Sections were deparaffinised, rehydrated to water and endogenous peroxidase activity quenched by placing the sections in 0.3% H_2_O_2_/methanol for 20 min at room temperature (RT). Following pressure cooker antigen retrieval in Access Super pH9.5 (Menarini Diagnostics, Wokingham, UK) and incubation with 1.5% normal serum for 30 min at RT, the sections were incubated with primary antibody (Table 2). To visualise antibody binding, the horse-radish peroxidase avidin biotin complex was used (Vectastain Elite goat IgG kit, Vector Laboratories, Peterborough, UK) with 3,3′-diaminobenzidine (DAB) as the chromagen (Vector Laboratories, UK; brown). Negative controls, either omission of the primary antibody or goat isotype control, were included in every run.

Dual labelling experiments to visualise colocalisation of NDRG2 with neurofibrillary tangles or GFAP were performed using a combined colour product method. Briefly, NDRG2 expression was first visualised as described above. Sections were then incubated with the avidin-biotin blocking kit (Vector Laboratories, UK), according to the manufacturer’s instructions. The tissue was incubated overnight at 4 °C with either anti-AT8 (1:400) or anti-GFAP (1:1000), followed by biotinylated secondary antibody, and visualised with the alkaline-phosphatase-conjugated avidin-biotin complex and alkaline phosphatase substrate 1 (Vector Laboratories, UK, red). Every immuno-run included a single-labelled section, which showed the same pattern and intensity of immunoreactivity as seen in the double-labelling experiments.

### 4.3. Quantitative Analysis of NDRG2

Assessment of NDRG2-specific immunoreactivity was performed by capturing bright-field microscopic images in 3 adjacent 350 μm-wide cortical ribbons, consisting of contiguous fields to cover the total cortical thickness through the apex of the gyrus, using a ×20 objective (Nikon Eclipse Ni-U microscope, Nikon Instruments Europe BV, Amsterdam, Netherlands) and analysed using the Analysis ^D software (Olympus Biosystems, Watford, UK). The image was thresholded and the immunoreactive area (%) of the field determined per total area examined.

### 4.4. Previously Assessed Astrocyte Markers and Age-Associated Pathology

Previous studies of the temporal cortex in the CFAS cohort using an identical approach have determined the immunoreactive profile (% area immunoreactivity) of astrocytes (GFAP, EAAT2, GS), microglia (CD68, MHC II) and AD pathology (Aβ, AT8) in 3 adjacent cortical ribbons [10,38,42]. To assess the DNA damage response, the total number of γH2AX positive astrocyte nuclei was quantitated using Analysis˄D software [38].

### 4.5. Statistical Analysis

As the data from all of the markers were positively skewed, non-parametric methods were used for statistical analysis. Differences between astrocyte markers were compared using Friedman’s test. For assessment in relation to Braak NFT stage, the cases were grouped into entorhinal stages (Braak stages 0-II; 30 cases), limbic stages (Braak stages III-IV; 50 cases) and isocortical stages of tangle pathology (Braak stages V-VI; 17 cases). Differences according to Braak group and to local plaque and tangle scores were assessed by Kruskal–Wallis test, and significance of trend assessed using the Jonckheere–Terpstra test (JT). Correlation analysis was performed using Spearman’s rank test. Statistical analyses were performed using the statistical package SPSS (version 23, IBM, Armonk, NY, USA).

## Figures and Tables

**Table 1:** Comparison of the immunoreactive profile (% area immunoreactivity) of the astrocyte markers NDRG2, GFAP, GS, and EAAT2 in the TCx of an ageing cohort.

Astrocyte Marker	NDRG2	GFAP	GS	EAAT2
Mean (SD)	16.46 (11.75)	8.49 (8.98)	0.76 (0.84)	29.94 (26.87)
Median (IQR)	14.43 (7.25–22.1)	5.79 (2.12–11.55)	0.51 (0.23–0.99)	23.11 (8.42–43.61)

**Table 2:** Antibody source and specificity.

Specificity	Isotype	Dilution	Antigen Retrieval	Supplier
AT8 (tau)	mouse IgG_1_	1:400	PC, AS pH9.5	Endogen, UK
CD68	mouse IgG_3κ_	1:100	MW 10 min, TSC	DakoCytomation, UK
EAAT2 (Glt-1)	mouse IgG_2A_	1:20	PC, AS pH9.5	Novocastra, UK
GFAP	rabbit IgG	1:1000	MW 10 min, TSC	DakoCytomation, UK
GS	goat IgG	1:500	PC, AR pH6.4	Millipore, UK
MHC II	mouse IgG_1κ_	1:20	MW 10 min, TSC	DakoCytomation, UK
NDRG2	goat IgG	1:75	PC, AS pH9.5	Santa Cruz, USA

MW: microwave; PC: pressure cooker; TSC: trisodium citrate buffer, pH6.5; MAR: Access Revelation pH6.4 (Menarini Diagnostics, UK); AS: Access Super pH9.5 (Menarini Diagnostics, UK).

## Abbreviations

Aβ	β-amyloid
AD	Alzheimer’s disease
CFANS	Cognitive Function and Ageing Study
CFAS	Cognitive Function and Ageing Neuropathology Study
CNS	Central nervous system
EAAT2	Excitatory amino acid transporter 2
GFAP	Glial fibrillary acidic protein
GS	Glutamine synthetase
NDRG2	N-myc downstream regulated gene 2
NFT	Neurofibrillary tangle
